# Technology-Based Feedback and Its Efficacy in Improving Gait Parameters in Patients with Abnormal Gait: A Systematic Review

**DOI:** 10.3390/s18010142

**Published:** 2018-01-06

**Authors:** Gema Chamorro-Moriana, Antonio José Moreno, José Luis Sevillano

**Affiliations:** 1 Department of Physiotherapy, Universidad de Sevilla, 41009 Sevilla, Spain; gchamorro@us.es (G.C.M.), toni.moreno.martin@gmail.com (A.J.M.); 2 Department of Computer Technology and Architecture, Universidad de Sevilla, 41012 Sevilla, Spain

**Keywords:** feedback technology, gait, rehabilitation, motor control

## Abstract

This systematic review synthesized and analyzed clinical findings related to the effectiveness of innovative technological feedback for tackling functional gait recovery. An electronic search of PUBMED, PEDro, WOS, CINAHL, and DIALNET was conducted from January 2011 to December 2016. The main inclusion criteria were: patients with modified or abnormal gait; application of technology-based feedback to deal with functional recovery of gait; any comparison between different kinds of feedback applied by means of technology, or any comparison between technological and non-technological feedback; and randomized controlled trials. Twenty papers were included. The populations were neurological patients (75%), orthopedic and healthy subjects. All participants were adults, bar one. Four studies used exoskeletons, 6 load platforms and 5 pressure sensors. The breakdown of the type of feedback used was as follows: 60% visual, 40% acoustic and 15% haptic. 55% used terminal feedback versus 65% simultaneous feedback. Prescriptive feedback was used in 60% of cases, while 50% used descriptive feedback. 62.5% and 58.33% of the trials showed a significant effect in improving step length and speed, respectively. Efficacy in improving other gait parameters such as balance or range of movement is observed in more than 75% of the studies with significant outcomes. Conclusion: Treatments based on feedback using innovative technology in patients with abnormal gait are mostly effective in improving gait parameters and therefore useful for the functional recovery of patients. The most frequently highlighted types of feedback were immediate visual feedback followed by terminal and immediate acoustic feedback.

## 1. Introduction

The basic motor functions of the human being, such as gait, can be altered because of a wide range of traumatalogical, neurological, rheumatic, etc. pathologies [1,2]. Hip arthrosis [3], knee osteoarthritis [4], strokes, hemiparesis [5,6,7], or lower-limb amputations [8], all produce important alterations to gait patterns.

Developments in technology and information technology (IT) have enabled the development of new techniques for gait re-training based on feedback supplied by electronic devices. This has been demonstrated by authors such as Druzbicki et al. [5], Basta et al. [9], Zanoto et al. [10] and Segal et al. [11].

The basic principle of feedback is the ability to voluntarily control and change certain bodily functions or biological processes when information is provided about them [12]. The main advantage of feedback is the supply of information about a specific biological process about which the patient does not consciously have information [13].

Currently, technology is developing towards facilitating the functional recovery of the patient, sometimes even without the physiotherapist. These treatments incorporate: robot assisted movement [10,14,15,16], virtual reality technology [17] and inertial monitoring devices [18,19] amongst others. Some of these systems use visual [5,11,20], acoustic [15,21] and/or haptic [22,23] feedback in a coherent and detailed way, adapted to each user’s individual needs [24]. New technologies based on feedback are extremely useful in the area of rehabilitation for re-educating an altered function or teaching a new one [2,25]. These aspects represent the main objectives of physiotherapy [13,25].

However, technological systems are frequently adopted in clinical practice without their efficacy having been proven. Researchers need to focus on providing clinical findings [24]. Therefore, the effects of these novel devices need to be measured [26,27] on different study populations, considering gait parameters, therapeutic guidelines adopted, clinical results obtained, systems of assessment used, etc. Similarly, we need to analyze the efficacy of different types of extrinsic feedback, in other words, that coming from an external source [28]. In this case, electronic devices will provide concurrent or immediate feedback, that is, feedback received simultaneously with the action (for example, during the foot support phase, the patient knows the amount of vertical reaction force of the floor on the limb or during walking the patient knows his/her speed); terminal or retarded feedback, or feedback received when the action is finished (for example, at the end of a tour the patient knows information about his/her progress, length of the steps, speed, kinematic of the knee, etc.); acoustic (e.g., beep, oral, etc.), visual (e.g., video cameras, displays, etc.) or haptic information (usually vibrations in some body area such as the soles of the feet) [29]; etc. Finally, this study also considers whether extrinsic feedback offers knowledge of performance (KP), in other words, characteristics of performance (e.g., if the foot bears the right direction, if the trunk remains erect during the action, etc.); or knowledge of result (KR) [30] (correct or incorrect action, score, etc.); whether this is descriptive (description of errors) or also prescriptive (how to correct errors) [24] (for example, we describe an error in walking saying that the patient is dragging the foot during the swing phase of the step. However, to correct it, we ask the patient to flex the hip and knee more when taking the step, so that the foot does not touch the ground).

Hence, the need to review, synthesize and analyze clinical findings related to the use of different kinds of technology-based feedback and their effectiveness in improving certain parameters in functional gait recovery.

## 2. Materials and Methods

The method was based on the PRISMA protocol [31].

### 2.1. Data Sources and Search Strategy

An electronic search of PUBMED, PEDro, WOS, CINAHL, and DIALNET was carried out from January 2011 to December 2016. In addition to this, we checked the reference lists of the included studies. Mesh terms (Medical Subject Headings) for English language or Decs Terms (Descriptores en Ciencias de la Salud) for Spanish database and search strategies are shown in Table 1 and Table 2.

### 2.2. Study Selection and Inclusion Criteria

The papers included in this review had to meet the following criteria:

- Population: Mainly patients with a modified or abnormal gait (i.e., spatiotemporal gait parameters) due to a pathology such as cerebral palsy, hip orthoprosthesis, lower member amputation, knee ligamentoplasty, etc.
- Interventions: application of technology-based feedback (haptic and/or visual and/or acoustic) to assist functional gait recovery as much as possible. The feedback had to be received by the patient directly (external feedback).
- Comparisons: Any comparison between different kinds of feedback (visual, haptic, immediate/concurrent, retarded/terminal, etc.) applied using technology. Or any comparison between technological and non-technological feedback, usual care or an alternative exercise therapy/intervention not based on feedback.
- Outcomes: Any validated measures of parameters or aspects associated to gait, such as: pain, functionality, balance, unload weight bearing, spatiotemporal parameters (speed, cadence, step length), kinematic data (range of movement-ROM) and score by specific gait assessment test or scale (i.e., Up and Go, chair-stand time).
- Study design: Randomised controlled trials (RCTs).
- Measure of methodological quality of RCT: A minimum of 4 points according to PEDro Scale. That is, “fair” and “high” quality studies [32] (see Quality Appraisal).
- Language: Studies reported in English or Spanish.
- Setting: Not limited to a particular setting.

The titles and abstracts of the search results were screened to check if a study met the pre-established inclusion criteria. We obtained the full text article of those studies which met the criteria, and documented the causes for any exclusions at this stage.

### 2.3. Data Extraction

Data extraction was carried out by one reviewer (A.J.M.) and checked for accuracy by a second reviewer (G.C.M.), using a table designed to detail information on study features, participant characteristics, feedback modality, technology employed (for feedback and assessment), interventions, comparisons, and outcome measurements.

### 2.4. Quality Appraisal

Apposite studies were assessed for methodological quality using the Physiotherapy Evidence Database (PEDro) critical appraisal tool [33]. This method was valid and reliable for assessing the internal validity of a study (criteria 2–9). We also evaluated the adequacy of the statistical information for interpreting the results (criteria 10–11) [34,35,36]. PEDro consists of 11 criteria overall; although criterion 1 refers to the external validity of the trial and is not included in the final score [34]. Each criterion could be Yes (one point) or No (0 points), with a maximum score out of ten. Only “fair” (scores 4/5) and “high” (scores ≥ 6/10) quality studies [32] were included in this review.

## 3. Results

### 3.1. Search Results

We found 884 articles in the electronic databases. Most of them in Pubmed (404), and the rest in PEDro (61), WOS (16), Cinahl (339) and Dialnet (64). Following the removal of duplicates, 776 articles were screened by title, abstract and full-text, due to: not including feedback technology, not applying the feedback directly to the patient, not being RCT, not using feedback for gait functional recovery, not having ≥4 score in PEDro Scale. After the screening, 20 studies were left for inclusion in this review.

**Figure 1:**
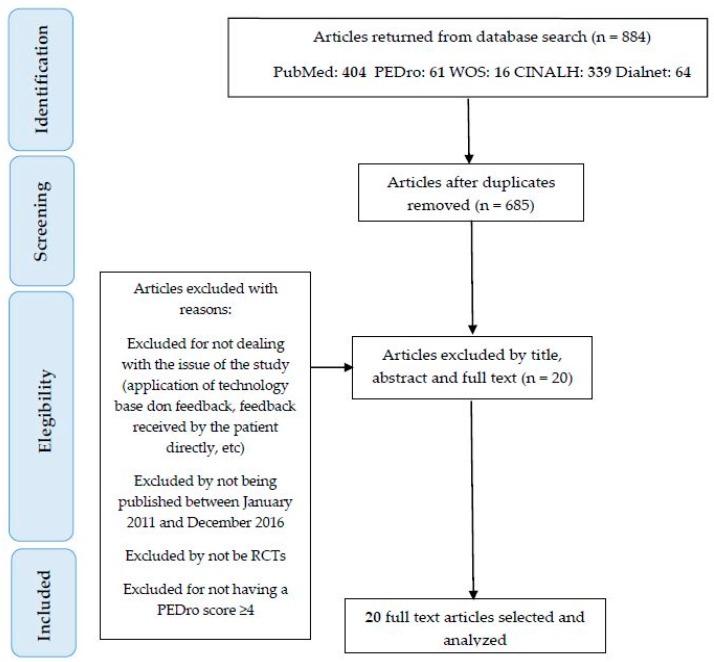
Research method of this study.

Figure 1 shows the search and study selection process, which was based on PRISMA [37] guidelines.

### 3.2. Characteristics of Included Studies

A detailed summary of the features and results of each selected study is shown in Table 3.

### 3.3. Quality Assessment

The results of the PEDro scoring are shown in Table 4. All the selected papers rated “fair” and “high” quality (≥4 points).

The item “Subjects were randomly allocated to groups” (2) was scored by all papers because it was an inclusion criterion. Besides, the items “Eligibility criteria were specified” (1) and “The results of between-group statistical comparisons are reported for at least one key outcome” (10) were scored in all studies apart from 2.

Although the studies were considered to be of “fair” and “high” quality, there were two items with 0 scores: “Blinding of all subjects” (5) and “Blinding of all therapists who administered the therapy” (6).

### 3.4. Participant Characteristics

Relative to the population in this review, neurological patients were found in 15 out of 20 papers (75%). That is: 8 of stroke [5,7,16,19,21,39,41,42]; 1 of cerebral palsy [17]; 2 of hemiparesis [14,18]; 4 of Parkinson’s [19,23,38,40]; and 1 with incomplete spinal cord injury [15]. Byl et al. [19] include stroke and Parkinson’s in the same research. Besides, 2 studies were found with patients in the orthopaedic area [11,20]; and 3 more with healthy subjects [10,22,26].

All participants were adults bar one [17].

### 3.5. Feedback Technology

Four studies [10,14,15,16] stood out due to their use of exoskeletons, although only 2 of them produced feedback, Alex II [10] and Lokomat [16]. The others used complementary technology which only assists gait: Gar [14] and Lokomat [15] in this case without feedback.

Six studies were based on load platforms [5,14,18,22,40,42], such as Smart Equitest^®^ [40], Gait Trainer^®^ [5,18] and Functional Trainer System^®^ [42]; and 5 on pressure sensors [11,19,22,26,39] for example Emed-Q100^®^ [39] or Ped-Alert TM120^®^ [21].

**Figure 2:**
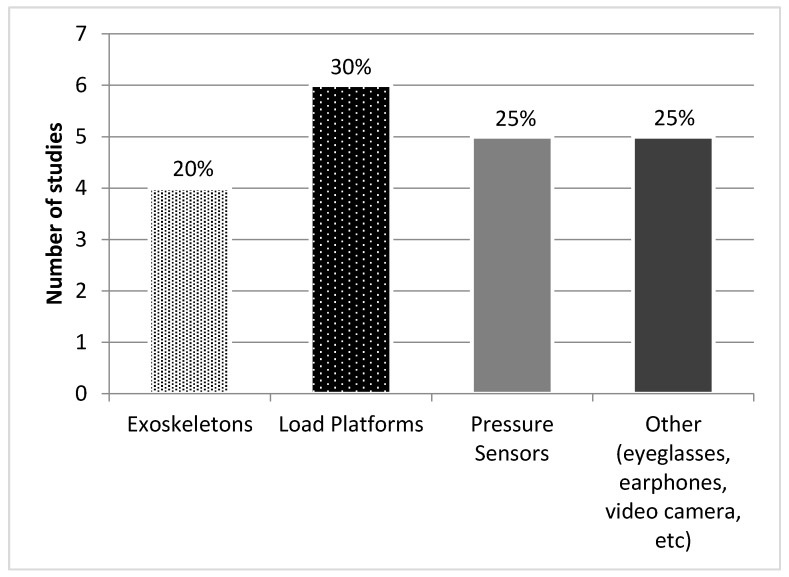
Feedback technologies.

The feedback technology was supplemented with other tools in 8 papers: treadmills [5,11,14,16,23,40], exoskeletons [14,15], forearm crutches [15], and metronome [18]. Figure 2 summarizes the use of technologies.

### 3.6. Feedback Modalities

The studies used different types of feedback: visual, acoustic and haptic; terminal/retarded and concurrent/immediate; descriptive and prescriptive; with both KR and KP. Visual feedback was used in 60% of the papers, acoustic in 40% and haptic in 15%. Terminal/retarded feedback was used in 55% and concurrent/immediate in 65%. Descriptive feedback was used in 50% of cases, with prescriptive in 60%. KP was featured in 45% and KR in 70% (Table 5).

**Figure 3:**
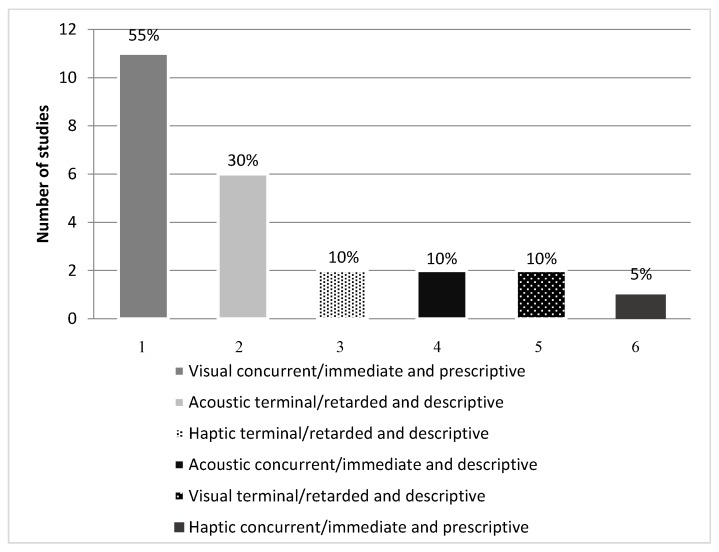
Types of feedback.

The combination of types of feedback used in descending order was: 55% visual, concurrent/immediate and prescriptive feedback [5,10,11,14,16,17,18,19,20,40,42]; 30% acoustic, terminal/retarded and descriptive [5,7,15,17,21,41]; 10% haptic, terminal/retarded and descriptive [22,26], acoustic, concurrent/immediate and descriptive [10,38] and visual, terminal/retarded and descriptive feedback [39,40]; 5% combined haptic, concurrent/immediate and prescriptive feedback [23] (Figure 3).

### 3.7. Assessment Technology

The technology used to assess gait in the selected studies was as follows: 3D movement analysis systems [5,18,20,23]; platform or treadmill force sensors [10,11,22,26,40]; pressure sensors in insoles [19], platforms [26] and parallel bars [7,15,21,22,40,41], pulsometer and ergospirometry [16]; functional training system [42]; exoskeleton [15]; and Gaitway [11].

### 3.8. Interventions and Comparators

In six studies the application of the feedback systems lasted 20 min [5,11,14,15,18,40], although some took up to 90 min [19]. Results also included some complementary treatments to technological feedback, such as balance [5], strength training [19], postural correction [23], stretching [7,40], speech therapy [16] and medications [11].

### 3.9. Outcome Measures and Results

The measurements taken in the studies were in descending order of frequency: speed, 75% [5,7,14,15,17,18,19,22,23,38,40,41]; step length, 50% [17,18,19,23,38,40,42]; Up and Go Test, 20% [19,21,22,41]; cadence, 20% [5,15,18,23]; ROM, 10% [18,23]; 10MWT 10% [5,42]; Berg Scale 10% [19,41] and 2MWT 10% [5,38]. Other parameters approached to a lesser degree were: IQR [5], peak respiratory rate [16], peak heart rate [16], etc.

For the most frequently considered parameters (speed, step length, Up and Go Test, Cadence, ROM, 10MWT and Berg Scale) the studies with significant outcomes were: 58.33% for speed [7,15,17,19,23,40,41]; 62.5% for step length [17,19,23,40,42]; 75% for TUG [21,22,41], 50% for cadence [15,23], 100% for ROM [18,23], 50% for 10MWT [42] and 100% for Berg Scale [19,41]. The clinical interventions of these studies with significant outcomes, except one [18], were effective in improving the parameters indicated. Table 6 summarizes these studies.

## 4. Discussion

The aim of this review was to synthesize clinical findings regarding the effectiveness of technological feedback in assisting functional gait recovery. Studies defending such effectiveness versus non-technological feedback include: Baram et al. [17], Ki et al. [21], El-Tamawy et al. [23] and Sungkarat et al. [41] amongst others. The authors of this study defend the use of technological feedback but not at the cost of usual care such as: mirror therapy [7], assisted gait [7] or verbal feedback [19], etc. In other words, technological feedback and traditional physiotherapy complement each other in assisting the functional recovery of the patient. To a lesser degree, other authors such as Brasileiro et al. [18], Byl et al. [19] or Hunt et al. [20], state that technological feedback did not obtain positive, or at least significant, results, in relation to other treatments.

In Physiotherapy, the current trend is to improve treatments using new technologies adapted as much as possible to the user needs. Furthermore, it is not only the system that must be individualized, but also the type of feedback used. To exemplify this trend, consider the GCH Control System [27], an instrumented forearm crutch that controls the loads exerted on the crutch when the patient has to partially discharge his/her affected limb. It includes a feedback mechanism to send information about these loads to both the physiotherapist and the patient. When the patient has deficiencies in their coordination skills, the first sessions are usually started with indirect feedback. That is, the therapist receives feedback from the system and verbalizes it to the patient. The patient finds it easier to understand the information through the physiotherapist, who verbally adapts it to their individual conditions (e.g., “Load a little more”, “Try to keep that same load”, “Be careful that you load more with the right stick than with the left”, etc.). The system also has the possibility of adapting the type of feedback (immediate, delayed, visual, auditory, etc.) according to the user's needs. For instance, based on our experience, the use of immediate feedback is easier for the patient and leads to a faster but less lasting result, so it is used when the patient has fewer skills. The delayed feedback is, on the contrary, more complex for the patient and the results come later, although they are more durable [43]. On the other hand, in the case of the GCH System the visual feedback is much simpler than the auditory feedback, which can only be used when the user completely dominates the former.

The articles analyzed in this review highlight how the feedback used when the subject is healthy is more complex [10,22] than when he/she is sick [7,15,17]. Also, in the present review, it is observed how there are parameters such as the cadence that can be easily corrected by means of a sound signal such as that emitted by a digital metronome or a more complex one by means of an exoskeleton [15,21,41]. On the other hand, deviations from the center of gravity are better worked by means of images [11,25].

However, it is worth mentioning that, again according to our experience, current technological systems have the tendency to personalize their treatments but without even nuancing the exact needs of the patient. It will be the therapist who makes the decision to use the technology in one way or another, always based on an initial and continuous assessment of the process and taking into consideration the coordinating, proprioceptive abilities of the user. The feedback received by the therapist for decision-making will be not only through technological means, but also through observational analysis. Both assessments, the technological and the visual or manual, are again complementary in the process of functional recovery of gait.

The technological devices, based on feedback, used by the different authors range from the complex to the basic. The complex group would include, for example: Biodex [5,18], Gaitway [11], GAR [14] or LOKOMAT [15]. The specific characteristics of each device means they each have pros and contras in terms of functionality. For example, LOKOMAT requires much more preparation time than GAR [14]. The basic devices include: heel switches [23], virtual glasses (used as computer monitor) and headphones [17], or a cane with a step-counting sensor [7]. The latter has been rendered obsolete as it has been superseded by other canes [27,44] with much more advanced technology and functions. These devices even have their own software designed specifically for functional gait recovery [27].

On the other hand, the high cost of these devices means that their everyday use is unfeasible despite their effectiveness [20]. Many authors [10,20,26], including those writing this article, favour efficiency versus the effectiveness of clinical technology in relation to financial, spatiotemporal and human resources [45]. In other words, clinical professionals require assessment and treatment systems which are feasible for everyday clinical practice, allowing adequate development of a process of functional [1,22] gait recovery. For instance, Quinzaños et al. [15] highlight the efficacy of the acoustic stimulus for re-training gait cadence and symmetry. As a result, a basic metronome [18] can be highly useful for functional gait recovery.

As this paper’s introduction shows, there are many different classifications of feedback. For example, depending on the sense used, it will be acoustic, visual or haptic [28]. Relating to the moment of the stimulus, there is immediate/concurrent or retarded/terminal feedback. Finally, if the information provides data about performance or result we would be talking about KP or KR [30]. The results of this review show that authors do not just use one isolated type of feedback, instead they sometimes prefer to combine them. The one used most on its own is visual feedback [5,10,11,14,16,17,18,19,20,39,40,42], which is also concurrent [5,10,11,14,16,17,18,19,20,23,38,40,42]. In contrast, combined, we find four articles with visual and acoustic feedback at the same time [5,10,17,38]: prescriptive and again concurrent visual feedback; and descriptive, concurrent or terminal, acoustic feedback. Summing up, of the RCTs selected in this review, 55% of the articles featured prescriptive and concurrent visual feedback [5,10,11,14,16,17,18,19,20,40,42], and 30% descriptive and terminal acoustic feedback [5,7,10,15,17,21,41]. Although many of the devices used in the clinical trials had more types of feedback available (for example, haptic [23,26]), the authors opted for concurrent feedback, either terminal acoustic or concurrent visual which are the most effective according to Agresta et al. [6]. Thus, it has been demonstrated that concurrent feedback produces the best short-term results [24], while retarded feedback obtains the best results in the long-term [46,47]. However, other authors such as Parker et al. [24] or Salmoni et al. [48] stress that feedback can be counterproductive for learning a complex task if the procedure is applied in too detailed a manner. In other words, detailed feedback can make it more difficult for the participant to understand or process other sensory information.

We must clarify that this statement refers especially to short-term learning, particularly if complex information is offered to patients with limited coordination skills. If we consider a long-term learning the patient has more time to assume complex information although the authors of this study advocate the progression in difficulty based on a continuous assessment of the process. Another handicap of complex and prolonged feedback is the creation of the patient's dependence on receiving feedback. In this sense, the patient responds to feedback automatically in a specific task but does not integrate the learning so it is unable to extrapolate it to other similar tasks [49].

On the other hand, all the information received by the patient can be descriptive (it simply states and describes the error) or prescriptive (it provides data on how to correct the error) [24]. When the correction is simple like in the aforementioned case of the instrumented forearm crutch, just by describing the load exerted the patient knows that he/she must exert more or less force. In other cases, the description and prescription of the correction are not so obvious. When a patient touches the ground with the foot in the swing phase of a step, the correction depends on the cause and this is multifactorial (kinematics, poor coordination, etc.). The patient may not flex the hip, knee or ankle sufficiently, either due to joint limitation or muscle weakness of the tibialis anterior in the case of dorsiflexion of the ankle, hamstrings for knee flexion or iliopsoas and anterior rectus of the quadriceps in the case of the hip. Another cause would be the lack of proprioception of the patient that prevents her/him from making the gesture or even carrying it out simultaneously (step and triple flexion of the lower limb at a time). In this case, the prescription must be offered by the physiotherapist based on the causes, in a progressive and individualized manner. Selective muscle strengthening exercises, manual therapy to gain range of motion in some joint or working the patient’s balance independently to the walking session may be prescribed.

Another example is arm movement during gait. Error detection and description can be easily implemented using technology. On the contrary, the prescription for its correction is usually more complex because again the causes are multiple: lack of integration of the arms in the body scheme, lack of dissociation between the scapular and pelvic waists, lack of mobility of the glenohumeral joint, etc. Deepening further, the patient can brace but not fluidly, i.e., without rotation of the shoulder girdle and without transferring the energy from proximal (trunk) to distal (arms), which would be incorrect. Even the patient may not swing arms in an opposing direction with respect to the lower limb, which would lead to an erroneous walking. Again the prescription must be made by the physiotherapist based on the cause and of course on a rigorous initial and continuous assessment.

Other authors such as Sigrist [49] affirm that to provide the idea of a movement, the feedback should be in principle prescriptive. Eventually, when the subject has internalized the action, descriptive feedback may be applied to make the correction more effective. Similarly, Sulzenbruck [50] states that, before the skill is acquired, prescriptive feedback is more effective than descriptive feedback. Still, there are authors such as Ki et al. [21] who use descriptive feedback (a beep to indicate that the weight load has been exceeded in the paretic limb) while others such as Segal et al. [11] opt for prescriptive feedback in his RCT (a graphic representation of the subject by means of a skeleton, on a screen, informs him how the optimal knee movement should be made).

Overall, the selected articles obtained significantly positive results in relation to the use of technological feedback. Even so, it should be noted that some specific parameters were not particularly significant. That is the case of stride speed or time [5,14,17,18,22,40], which can be influenced by complex robotized systems or exoskeletons, treadmills, supports etc., and the focus of the user’s attention on other parameters of interest. These show an improvement in overall gait despite not actually increasing speed.

As for the populations covered, most of the technological feedback applications were applied in the neurological field. The results of this review show that 75% came from that area [5,7,14,15,16,17,18,19,21,23,38,39,40,41,42]. Hence, feedback is capable of changing motor strategies in patients with neurological lesions [18], with the application of this type of treatment being more appropriate during early stages of rehabilitation [24]. As for other clinical areas, this review has only included 2 articles (10%) based on muscular-skeletal lesions [11,20]. They outlined the limitation of traditional physiotherapy in the recovery of lower-limb functions [51]. Only 3 articles (15%) used a sample of healthy subjects [10,22,26]. Despite being an RCT, it is sometimes necessary to perform research with healthy subjects to ascertain the efficacy of a new technological system before using it with patients requiring treatment. Continuing with the study population, it should be noted that 95% of the reviewed articles included samples of adult subjects [5,7,10,11,14,15,16,18,19,20,21,22,23,26,38,39,40,41,42]. Only 5% of the subjects were under 18 [17]. For this reason, we believe more scientific findings need to be generated in other clinical areas and in young population samples.

The following gait parameters were assessed in the selected RCTs, in descending order of frequency: speed (cm/s) [7,15,17,18,19,22,23,38,40,41], step length (m) [15,17,18,19,22,23,38,41,42], and cadence (steps/min) [5,15,18,23]. These parameters were chosen because the unit of gait is the step and time-space parameters are essential for its assessment [2,52,53,54,55]. The measurement devices were in some cases also those providing the feedback [7,10,14,19,26,39,40,41,42]. The majority measured short-term effects [5,7,14,15,16,18,19,20,21,23,41,42]. The few which measured long-term effects did not obtain conclusive results [11,39,40], which underlines the need for prospective studies.

As a final reflection, the authors of this study recognize that technological progress has led to the development of highly useful tools in the field of physiotherapy which complement conventional therapy. In no case are these technologies considered substitute media, in contrast to the opinion of Parker et al. [24]. Despite the multiple benefits which new technologies offer, a physiotherapist’s face-to-face treatment of a patient cannot be equaled by technological means. The personalized and intuitive adaptation of the health-care professional is the key to successful treatment.

## 5. Conclusions

Treatment based on feedback using innovative technology in patients with abnormal gait is mostly effective in improving gait parameters and therefore of use in the functional recovery of a patient.

Concurrent/immediate visual is the most frequently used type of feedback, followed by terminal/retarded acoustic. Also, prescriptive feedback and knowledge of result are the most frequent alternatives.

Most of the systems used are based on force and pressure sensors, normally accompanied by complementary software.

Walking speed is the most frequently evaluated parameter, with the majority of studies reporting significant improvements (in one study the changes were only significant after 3 months). The positive effect on the stride length is also found significant in most cases. In general, the number of studies with significant outcomes for the other parameters (such as balance or range of movement) is too low.

## Figures and Tables

**Table 1:** Mesh and Decs Terms put into groups by mean.

Terms and Strategies	Identifier
feedback or biofeedback or neurofeedback or proprioception	1
treatment or program * or exercise * or rehabilit * or training or educat * or “stimulation training” or teaching or learning	2
software or program * or technology or “biomedical technology” or system	3
gait or walking or ambulation or locomotion or “stair navigation”	4
Randomiza * or study or “clinical trial”	5
Trata * or program * or rehabilit *	6
feedback or biofeedback or neurofeedback or retroalimentación	7
marcha or ambul * or locomoción	8

**Table 2:** Search strategy.

Database	Search Strategy	Simplified Strategy
PubMed	(treatment or program * or exercise * or rehabilit * or training or educat * or “stimulation training” or teaching or learning) and (feedback or biofeedback or neurofeedback or proprioception) and (gait or walking or ambulation or locomotion or “stair navigation”) and (software or program * or technology or “biomedical technology” or system)	2 and 1 and 4 and 3
PEDro	feedback and gait	1 and 4
WOS	(feedback or biofeedback or neurofeedback or proprioception) and (gait or walking or ambulation or locomotion or “stair navigation”) and (software or program * or technology or “biomedical technology” or system) and (randomiza * or study or “clinical trial”)	1 and 4 and 3 and 5
CINAHL	(feedback or biofeedback or neurofeedback or proprioception) and (gait or walking or ambulation or locomotion or “stair navigation”)	1 and 4
Dialnet	(trata * or program * or rehabilit *)and (feedback or biofeedback or neurofeedback or retroalimentación) and (marcha or ambul * or locomoción)	6 and 7 and 8

**Table 3:** Characteristics of included studies.

Study Characteristics	Participant Characteristics	Feedback Technology/Feedback Modality	Intervention and Comparison	Outcomes Measurements
1. Baram, Y.; 2012 [17] To study the effects of gait training with visual and auditory feedback cues on the walking abilities of patients with gait disorders due to cerebral palsy (CP)	N ^1^ = 35 Sex = 20 female (57.14%); 15 male (42.85%) Age = 12.2 ± 6.2 years Inclusion criteria: not specified Exclusion criteria: not specified	Eyeglasses with virtual reality/Visual prescriptive and concurrent (KP ^2^) Earphones with a clicking sound/Acoustic descriptive and terminal (KR ^3^) Assessment Technology: Accelerometer	CG ^4^ visual (n = 7)/CG auditory (n = 8): healthy individuals walk on a 10 m track without technological assistance. IG ^5^ visual feedback (n = 10): CP patients walk on a 10 m track with transversal lines (virtual reality) which change according to gait. IG auditory feedback (n = 10): CP patients walk on a 10m track while a “clip” is heard at each step. Frequency and duration: measurement before exercise without device, after 20´exercise and after 20´rest and again without the device.	Average Improvement (95% *CI* ^6^): IG visual feedback: Significant and effective (*): Walking Speed (m/s), Stride Length (m) Not significant: None IG auditory feedback: Significant and effective: Walking Speed (m/s) Not significant: Stride Length (m)
2. Brasileiro, A. et al., 2015 [18] Immediate effects of visual/auditory biofeedback, combined with partial body weight supported (PBWS) treadmill training on the gait of people with chronic hemiparesis	N = 30 Sex = 12 female (40%); 18 male (60%) Age = 56.4 ± 6.9 years Inclusion criteria: chronic stroke status with hemiparesis, capable of walking with assistance or auxiliary aparatus, low or moderate velocity, free cognitive capacity Exclusion criteria: other visual and/or auditory neurological and orthopedic pathologies, hypertension during performance, not understanding instructions	Gait Trainer^®^ System 2 y Biodex Unweighting System/Visual prescriptive and concurrent (KP) ^a^ Metronome Assessment Technology: 8-camera based motion capture system at 120 MHz with tracking markers located at the pelvis, thigh, leg and foot	CG (n = 10): gait training with parallel bars IG I (n = 10): idem + partial unweighting system and visual feedback for symmetry and stride length IG II (n = 10): idem + partial unweighting system and an acoustic stimulus (“beep” to a cadence of 115%) Frequency and duration: sessions of 20 min, two minute rest until heartbeat frequency reaches 75%	Pre-test vs. Post-test (95% CI): Spatiotemporal gait variables: Significant and effective: None Not significant: Speed (m/s), Stride length (m), Cadence (steps/min) Angular gait variables: Significant and effective: None Significant and not effective: Range Of Motion (ROM) Hip (°), ROM ankle (°) Not significant: ROM Knee (°)
3. Byl et al., 2015 [19] evaluate if visual and kinematic feedback provided during supervised gait training would interfere or enhance mobility, endurance, balance, strength and flexibility in older individuals more than one year post stroke or Parkinson’s disease(PD)	N = 24 Sex = 13 female (54.2%); 11 male (45.8%) Age = 30–75 years Inclusion criteria: abnormal gait one year after stroke or Parkinson’s; speak English or use an interpreter; able to follow instructions; motivation and ability to walk a minimum of 100 steps Exclusion criteria: Not specified	iPad^®^ with program LabVIEW/Visual prescriptive and concurrent (KP) Assessment Technology: Pressure sensors (shoe pad) Joint angle sensors (accelerometer, magnetometer and gyroscope)	CG (n = 12): conventional gait therapy (stairs, fitball, theraband, etc.) IG (n = 12): idem + visual cinematic feedback Frequency and duration: from 6 to 8 weeks with an average of 12 sessions of 90 min each. Encouraged to walk and take part in activities of daily living (ADL)	Post exercise–baseline difference scores: CG compared to IG (*ES* ^7^) Significant and effective: Gait Speed—10 m walk (m/s), Step length (m), Tinetti Score, Berg Balance, Strength (lbs) (affected), Strength (lbs) (unaffected), ROM (deg) (affected), ROM (deg) (unaffected) Not significant: 6 min walk (cm), Five Times Sit to Stand (FTSTS) Test (s), Timed Up and Go (TUG) (s)
4. Druzbicki, M. et al., 2015 [5] Effects of gait training using a treadmill with and without visual biofeedback in patients in the late period after stroke, and to compare both training methods	N = 50 Sex = 18 female (36%); 32 male (64%) Mean Age = 62 years (range 38–79 years). Inclusion criteria: ischemic stroke, minimum 6 months walking, without rehabiliation for at least 6 months, autonomous gait, Brunnstrom Scale: 3–4, Ashworth ≤ 1 (lower limb musculature), Rankin Scale (disability): 3 Exclusion criteria: unstable hemodynamics, peripheral vascular disease, Mini Mental Test < 20 (cognition), significant gait disorders	Gait Trainer^®^ System 2/Visual prescriptive and concurrent (KP) Signal confirming correct execution/Acoustic descriptive and terminal (KR) ^a^ Treadmill Assessment Technology: SMART de BTS Bioengineering (6-camera based system at 120 MHz with tracking markers located at the sacrum, pelvis, femur, fibula and foot)	CG (n = 25): conventional physiotherapy and treadmill program (balance, active and breathing exercises) IG (n = 25): idem + visual feedback (locates the position of the foot and where it should go) Frequency and duration: 15 to 20 min on the treadmill, 1 ½ hour sessions for 10 days plus two weeks of basic physiotherapy	Baseline—post-exercise Significant and effective: Stance phase of the non-paretic limb (STF_np_) (% of cycle), Swing phase of the non-paretic limb (SWF_np_) (% of cycle), Lenght of the cycle of non-paretic limb (LC_np_) (%) Not significant: Cadence (steps/min), Velocity (m/s), Stance phase of the paretic limb (STF_p_) (% of cycle), Swing phase of the paretic limb (SWF_p_), Length of the cycle of paretic limb (LC_p_) (%), 10-m walk test (10MWT) (m/s), 2-min test (m), Test Up and Go (TUG) (s)
5. El-Tamawy et al., 2012 [23] To determine the influence of paired proprioceptive cues on gait parameters of individuals with Parkinson´s Disease (PD)	N = 30 Sex = 9 female (30%); 21 male (70%) Age = 62.5 ± 6.1 years Inclusion criteria: walk independently for 6 min continuously on the treadmill, United Parkinson´s Disease Rating Scale (UPDRS) ADL/motor: light-moderate disability, diagnosed 3–5 years earlier, stable medication, ability to give informed consent Exclusion criteria: another gait-altering neuro-muscular-skeletal disorder; uncontrolled blood pressure, cardiovascular disease	Pressure sensor in the toe (OPTEC Co. Ltd., Japan)/Haptic prescriptive and concurrent (KR) ^a^ Treadmill Assessment Technology: Qualysis ProReflex movement capture (Qualysis Medical AB, Sweden)	CG (n = 15): conventional treatment (stretching, balance, transfers, etc.) IG (n = 15): Proprioceptive neuromuscular facilitation techniques + vibratory feedback (are activated when the foot is in the swing phase) + conventional treatment Frequency and duration: 3 sessions per week for 8 weeks. CG: sessions of 45´; IG: sessions of 51´–70´	Baseline—Post-exercise Spatiotemporal Parameters of Gait: Significant and effective: Cadence (step/min), Stride Length (m), Walking Speed (km/h), Walking Distance (km) Not significant: None Lower Limb Angular Excursion: Significant and effective: Hip Flexion (°), Knee Flexion (°), Ankle Dorsiflexion (°) Not significant: None
6. Fu, M.C. et al., 2014 [26] To assess a novel method of using real-time haptic (vibratory/vibrotactile) biofeedback to improve compliance with instructions for partial weight bearing	N = 30 Sex = 14 female (46.7%); 16 male (53.3%) Age = 22 to 32 years Inclusion criteria: good health, walk without assistance, coordination and strength of upper limbs for walking with sticks Exclusion criteria: restriction in lower limbs for bearing weight and impossibility of using sticks	Haptic feedback belt with 3 vibration motors (axle-less vibration motors Pololu 10 mm P/N 1636) + Processing unit (Arduino Nano, Italy) to know the moment at which to apply the feedback/Haptic descriptive and terminal (KR) Assessment Technology: Force plate with 4 pressure sensors in a boot (Sparkfun Electronics, SEN-10245) SmartStep System (for the dynamic validation of the system)	Participants instructed to unload lower limbs 25 lb (range accepted from 15 to 35 lb). Forearm crutches and systems of sensors are used. “Haptic Biofeedback” Training Group (GFB) (n = 10): receive vibrotactile signal if acceptable range is exceeded “Verbal Instruction” Training Group (GCV) (n = 10) “Bathroom Scale” Training Group (GCB) (n = 10) Frequency and duration: first take 50 practice steps	Comparison between GCV, GCB y GFB Significant and effective: Load on the boot (lb), Percentage of participants’ body weight (%) Not significant: None
7. Ginis, P. et al., 2016 [38] Pilot RCT. To test the feasibility of training with a smartphone application (CuPiD system) in the home environment, and to discover the differential effects of CuPiD training versus conventional home-based gait intervention on gait, balance and health-related quality of life (HR-QoL) in Parkinson´s Disease (PD)	N = 38 Sex = Not specified Age = Not specified Inclusion criteria: walk for 10 min continuously; score of 24 or higher on Montreal Cognitive Assessment (MoCA); Hoehn & Yahr Stage II to III in ONstate and stable PD medication Exclusion criteria: severe medical conditions affecting gait other than PD, hearing or visual problems precluding benefiting from auditory feedback and likely to change medication regime during the course of the study.	CuPiD system: Smartphone (Galaxy S3-mini, Samsung, Korea) Docking station Two inertial measurement units (IMUs) (EXLs3, EXEL, srl., Italy) Applications used in this study: Instrumented cueing for freezing of gait training (FOG-cue app) Audiobiofeedback (ABF-gait app)/Acoustic descriptive and concurrent (KP) Assessment Technology: Earphones or smartphone´s speaker	CG (n = 18): to walk without feedback devices IG (n = 20): idem + feedback devices (ABF-gait app + FOG-cue app) Frequency and duration: 30 min walking, 3 sessions/week for 2 weeks	Pre-test vs. Post-test Not significant: Gait speed (m/s), Stride length (m), Four Square Step Test (FSST) (s), 2 Minute Walk Test (2MWT), MiniBESTest (0–32), Physical Activity Scale for the Elderly (PASE) (0–400), Unified Parkinson’s Disease Rating Scale (UPDRS) (0–132), New-FOG questionnaire (NFOG-Q) (0–28), Ziegler protocol (0–36), Falls Efficacy Scale-International (FES-1) (16–64), Short Form 36 (SF-36) (0–100)
8. Hunt et al., 2014 [20] Crossover. To compare performance error and perceived difficulty during toe-out gait modification in people with knee osteoarthritis (OA) across three different types of visual feedback: mirror, raw video, and real-time biofeedback of toe-out angle	N = 20 Sex = 11 female (55%); 9 male (45%) Age = 65.4 ± 9.8 years Inclusion criteria: knee OA Exclusion criteria: replacement of lower limb joints, knee surgery or injections in the previous six months, rheumatoid arthritis, arthrosis in other lower limb joints, inability to walk on a treadmill unassisted for 15 min	Video camera placed directly in front of the participant/Visual prescriptive and concurrent (KP) Assessment Technology: A Motion Analysis Corporation’s motion capture system consisting of 10 capture cameras at 120 Hz and 22 passive reflective markers	Participants were trained to gait on treadmill to increase the divergence 10° during stance phase by comparison with convergence angle during the selected gait Stage A: Mirror positioned 3 m in front of the participant (with a green line depicting the target angle) Stage B: Video screen positioned 3.2 m in front of the participant, overlaying the raw video image of the foot with a green tape target Stage C: The same video screen, but streaming real-time toe-out angle (a thin black line) and a green tape target Frequency and duration: 2´–3´ to become familiar with the tool and 15´´ to record data	Results measured after the intervention (raw video vs. mirror vs. real-time feedback) Significant and effective: Toe-out error (°) Not significant: Perceived difficulty (0–10)
9. Jung et al., 2015 [7] Effect of gait training when using a cane with an augmented pressure sensor for enhancement of weight-bearing over the affected lower limb on the peak pressure force of the cane, muscle activation & gait in patients with stroke	N = 21 Sex = 7 female (33.3%); 14 male (66.7%) Age = 56.4 ± 11.1 years Inclusion criteria: first unilateral stroke, Mini Mental Test ≥ 24, capable of walking with a cane, bearing more than 7% of body weight with cane in vertical position Exclusion criteria: cerebral aneurysm, hemianopia, dizziness, or other symptoms indicating vestibular impairment, impaired touch and pressure sensation on the non-affected hand, hemineglect, orthopedic disease influencing gait	Presssure sensor (CD 210-K200, Dacell Co. Ltd, Cheongju, Korea) and indicator (DN30W, Dacell Co. Ltd, Cheongju, Korea)/Acoustic descriptive and terminal (KR) Assessment Technology: Specific instrumented cane for this study GAITRite walkway system (CIR Systems Inc., Franklin, NJ, USA) + Surface electromyography (Telemyo 2400 G2, Telemetry EMG system, Noraxon, Scottsdale, AZ, USA) for gluteus medius and vastus medialis	CG (n = 10): gait training + conventional therapy IG (n = 11): idem + acoustic feedback (a beep is emitted when a weight above the threshold is borne) Frequency and duration: 30 minute sessions, five times a week for 4 weeks	Results measured pre-test vs. post-test (Mean difference, 95% *CI*) Significant and effective: Vertical Peak Force of the cane (% body weight), Muscle Activation (% non-paretic peak activity) gluteus medius and vastus medialis oblique, Single Support Phase of the affected side (% Gait Cycle), Walking Velocity (cm/s) Not significant: None
10. Khallaf et al., 2014 [39] To investigate the effect of task specific exercises, gait training, and visual biofeedback on correcting equinovarus gait among individuals with stroke	N = 16 Sex = 4 female (25%); 12 male (75%) Age = 40.8 ± 2.89 years Inclusion criteria: first unilateral stroke, hemiparesis minimum 3 months, medically stable, capable of understanding the procedure and giving informed consent, Chedoke-McMaster Stroke ≥ stage 4 (motor recovery), Modified Ashworth Scale(MAS): spasticity < 2, capable of walking autonomously with or without assistance for 6´ Exclusion criteria: altered sensation; cognitive, mental and visual deficiency; contractures in ankle and knee; taking muscle relaxant	Pedography (Colored graphs simulating foot placement) + emed-q100 pressure platform with 6080 sensors over a sensor area of 475 × 320 mm² and resolution of four sensors/cm^2^ at 100 Hz./Visual descriptive and terminal (KR) Assessment Technology: A capacitance-based pressure platform (emed-q100, Novel GmbH, Munich, Germany) was used for detecting the Pattern of foot placement	CG (n = 8): programme of conventional physiotherapy (strengthening exercises for the foot evertors and ankle dorsiflexors in addition to prolonged stretching of the calf muscles, walk in parallel bars and solid ankle foot orthosis (AFO)) IG (n = 8): specific exercises(stretching, musclespecific progressive-resistive exercise, balance training, etc) + gait training + visual biofeedback Frequency and duration: 5 sessions per week for 8 weeks, 50 min for each session	Results measured Baseline vs. postintervention vs. one month after intervention Time of Contact (percentage average rollover period): Significant and effective: Hindfoot, First Metatarsal Head, Second Metatarsal Head, Third Metatarsal Head, Forth Metatarsal Head, Fifth Metatarsal Head Not significant: None Maximum Force (N/cm^2^): Significant and effective: Hindfoot, First Metatarsal Head, Second Metatarsal Head, Third Metatarsal Head, Forth Metatarsal Head, Fifth Metatarsal Head Not significant: None
11. Ki et al., 2015 [21] To examine the effects of auditory feedback during gait on the weight bearing of patients with hemiplegia resulting from a stroke	N = 25 Sex = 6 female (24%); 19 male (76%) Age = 57.7 ± 10.75 years Inclusion criteria: Stroke minimum 6 months previously, mini-mental test ≥ 24, walk autonmously at least 10 m unassisted, no orthopedic aids Exclusion criteria: Not specified	Pressure meter Ped-AlertTM120 (ORBITEC, Madison, WI, USA)/Acoustic descriptive and terminal (KR) Assessment Technology: GAITRite (CIR Systems Inc, Franklin, NJ, USA) + software GAITRite GOLD, version 3.2b	CG (n = 13): walk on GAITrite without feedback + treatment of neurodevelopment IG (n = 12): idem + acoustic feedback (a beep every time 50% of the patient’s body weight was exceeded on the paretic leg) Frequency and duration: the training period was a total of 4 weeks	Pre-test vs. Post-test Significant and effective: Duration of the Stance Phase (%), Duration of the Single Limb Stance (%), TUG test (sec) Not significant: None
12. Lipsitz L.A. et al.; 2015 [22] Crossover. To test whether sub-sensory vibratory noise applied to the sole of the foot using a novel piezoelectric vibratory insole can significantly improve sensation, enhance balance, and reduce gait variability in elderly people, as well as to determine the optimal level of vibratory noise and whether the therapeutic effect would endure and the user’s sensory threshold would remain constant during the course of a day	N = 12 Sex = 11 female (91.7%); 1 male (8.3%) Age = 73.8 ± 8.1 years Inclusion criteria: age 65–90 years, sense the vibrations in the insole, speak English, understand and provide informed consent, follow instructions Exclusion criteria: feet ulcers, Parkinson’s or other neurodegenerative diseases, chronic pain in lower limbs avoiding standing or walking, no equilibrium without support for 1´, not feeling the vibration when the insoles are set to maximum, uncomfortable with insoles, new drug in the previous 30 days, having participated in another study in the previous 30 days, any other condition deemed inappropriate by the researchers	Two piezoelectric actuators in insolates/insoles (2.5 cm diameter each)/Haptic descriptive and terminal (KR) Assessment Technology: Force platform Type 9286B force plate (Kistler Instrument Corp., Winterthur, Switzerland) GAITRite; CIR Systems, Inc. + software MATLAB	The correct vibration threshold was determined. Then, the stimulation of each insole was set at 0%, 70% and 85% of the threshold value in accordance with randomization. The values were modified in the middle and at the end of the session to check them with the reference value	Mean for Each Stimulation Level (95% *CI*) Significant and effective: TUG test (sec), Stride Time, left foot (sec) Not significant: Gait speed (cm/s), Stride Time, right foot (sec), Step Width (cm), Double Support (sec)
13. Ochi et al., 2015 [14] To examine whether gait training with a gait-assistance robot (GAR) improves gait disturbances in subacute nonambulatory hemiplegic stroke patients more than over-ground conventional gait training	N = 26 Sex = 6 female (23.1%); 20 male (76.9%) Age = 63.65 ± 9.8 years Inclusion criteria: first stroke less than five weeks prior to the study, unilateral hemispheric brain damage confirmed by computed tomography (CT) or magnetic resonance imaging (MRI), age 40–85 years, serious palsy of lower limbs (level III), Functional Ambulation Classification (FAC) ≤ 2, autonomous gait before stroke, informed consent Exclusion criteria: height < 145 cm or > 180 cm, body weight ≥ 100 kg, marked limitation in ROM of lower limbs, cardiovascular, respiratory, kidney or muscular-skeletal illnesses, difficult communication	Load sensors inserted between the sole of the foot and the foot bed of the shoe (the visual feedback regarding the stance phase and load amount)/Visual prescriptive and concurrent (KR) ^a^ GAR (Gait-assisted robot) ^a^ Treadmill	Overground conventional gait training group (OCGT) (n = 13): physiotherapeutic treatment (ROM and muscle strengthening exercises), speech therapy and occupational therapy + OCGT therapy (gait with parallel bars with orthesis of knee–ankle) and gait without parallel bars using forearm crutches) GAR-assisted gait training group (GAGT) (n = 13): idem (except OCGT) + GAGT therapy (lights for the foot pressure biofeedback system). Frequency and duration: 5 days per week for 4 weeks. Session of 60´ for physiotherapy, 60´ for speech therapy and 60´for occupational therapy and 20´ for GAGT or OCGT therapies	Pre-test vs. Post-test Significant and effective: Functional Ambulation Classification (FAC), Functional Independence Measure (FIM™) mobility score Not significant: walking Speed (m/s)
14. Quinzaños Fresnedo, J.; 2015 [15] Short-term effect of gait training of robotic orthoses with auditory feedback in patients with chronic incomplete spinal cord injury	N = 33 Sex = 24 female (77.4%); 7 male (22.6%) Age = 35.6 ± 16.4 years Inclusion criteria: Age: 18–65 years, hospitalized at National institute of Rehabilitation with incomplete spinal cord injury, American Spinal Injury Association (ASIA) scale: C-D, independent gait with technical help more than 6 months, informed consent Exclusion criteria: Not specified	Metronome Zoom GFX707II GuitarMulti-Effects Pedal (Zoom Corporation, Tokyo, Japón)/Acoustic descriptive and terminal (KR) ^a^ Forearm crutches ^a^ Walker Assessment Technology: Lokomat^®^ (Hocoma, Volketswil, Suiza) GAITRite^®^ System mat (CIR Industries, Clifton, NJ, USA)	CG (n = 16): functional recovery of the conventional gait IG (n = 17): Idem using Lokomat^®^ (auditory feedback) Frequency and duration: 12 sessions of 20´, 4 sessions per week for 3 weeks	Post-test (CG vs. IG) Significant and effective: Walking Speed (cm/s), Cadence (step/min), Stride Left (cm), Stride Rigth (cm), Functional Ambulatory Profile (FAP) Not significant: None
15. Segal, N.; 2015 [11] To determine whether individualized gait training is more effective than usual care for reducing mobility disability and pain in individuals with symptomatic knee osteoarthritis	N = 48 Sex = 32 female (66.67%); 16 male (33.33%) Age = 59.6 ± 6.4 years Inclusion criteria: activities of daily living (ADL) ≤ 9, > 18 years old, gait without help and to climb 2 steps, surgery more than 6 months prior to study, symptomatic knee osteoarthritis Exclusion criteria: amputation, severe back pain, serious heart or neurological illness, surgery in the previous 6 months, corticosteroid injections in the previous 3 months	Software (C-Motion, Inc., Germantown, MD, USA) + Optotrack, Model 3020 (force plate + 3D viewing system)/Visual prescriptive and concurrent (KR) ^a^ Treadmill Assessment Technology: Gaitway, h/p/cosmos sports & medical gmbh, Nussdorf-Traunstein, Germany Kistler Force plate Model 9286 with capture at 300 Hz	CG (n = 19): Conventional approach (use of pain medications for knee symptoms, knee surgery, and/or physical therapy) IG (n = 29): idem + gait training on treadmill by feedback to optimize movement of knees (skeleton model and target area) (the major goals in retraining gait were to move participants toward symmetrical and typical displacements of the trunk and pelvis about neutral frontal (x) and transverse (y) axes) Frequency and duration: 2 sessions per week for 3 months, session of 45´ for conventional treatment and 3 intervals of 8´ for training with feedback. 3–5´for resting and correction from physiotherapists	Post-test (CG vs. IG) (95% *CI*) After 3 months: Significant and effective: Late Life Function and Disability Index (LLFDI) basic lower limb function score, Knee Injury and Osteoarthritis Outcome Score (KOOS) symptoms, KOOS pain Not significant: Long Distance Corridor Walk (LDCW) time (sec), Chair-Stand Time (sec), Stair Climb Time (sec) After 6 months: Significant and effective: LLFDI basic lower limb function score, Chair-Stand Time (sec) Not significant: LDCW time (sec), Stair Climb Time (sec), KOOS symptoms, KOOS pain After 12 months: Significant and effective: Chair-Stand Time (sec), KOOS symptoms, KOOS pain Not significant: LLFDI basic lower limb function score, LDCW time (sec), Stair Climb Time (sec)
16. Shen, X.; 2014 [40] To explore whether balance and gait training with augmented feedback can enhance balance confidence in Parkinson´s Disease (PD) patients immediately after treatment and at 3–12 month follow-ups	N = 51 Sex = 20 female (39.2%); 31 male (60.8%) Age = 64.3 ± 8.25 years Inclusion criteria: Idiopathic Parkinson’s, stable medication, independent gait for 10 m, capable of following instructions (Mini-Mental Test > 23.19) Exclusion criteria: other neurological conditions, non-compensated cardiovascular disease, visual impairment, recent muscular-skeletal disorders in the back or lower limbs which alter gait and balance	KSD Technology Co Ltd., Shenzhen, China/Visual descriptive and terminal (KP) Smart-EquiTest Balance Master (NeuroCom International Inc., Clackamas, OR, USA)/Visual prescriptive concurrent (KP) ^a^ Treadmill Assessment Technology: GAITRite walkway (CIR Systems Inc., Havertown, PA, USA) Smart-EquiTest Balance Master (NeuroCom International Inc., Clackamas, OR, USA)	CG (active control group, CON) (n = 25): strength training of lower limbs (2 × 15 repetitions with 60% RM) IG (balance and gait training group, BAL) (n = 26): gait and balance training by visual and verbal feedback Frequency and duration: 12 weeks (eight in lab and four at home). Sessions of 60´, three sessions per week in lab; and sessions of 20´, five sessions per week at home	Pre-test vs. Post-test vs. Post-test (3 months) vs. post-test (12 months) Immediately after treatment: Significant and effective: Activities-Specific Balance Confidence (ABC) Scale (0–100), Movement velocity (°/s), Stride Length (cm) Not significant: End Point Excursion (Limit of Stability, LOS) (%), Gait Velocity (cm/s), After three months: Significant and effective: Activities-Specific Balance Confidence (ABC) Scale (0–100), Stride Length (cm), End Point Excursion (Limit of Stability, LOS) (%), Gait Velocity (cm/s), Stride Length (cm) Not significant: None After six months: Significant and effective: Activities-Specific Balance Confidence (ABC) Scale (0–100), Gait Velocity (cm/s), Stride Length (cm) Not significant: Movement velocity (°/s), End Point Excursion (Limit of Stability, LOS) (%)
17. Stoller, O. et al., 2015 [16] Pilot RCT. Efficacy and feasibility of feedback-controlled robotics-assisted treadmill exercise (FC-RATE) for cardiovascular rehabilitation in persons with severe impairments shortly after stroke	N = 20 Sex = five female (36%); nine male (64%) Age = 61 ± 11 years Inclusion criteria: First stroke less than 20 weeks prior to study, >18 years old, functional gait, understand the study and give informed consent Exclusion criteria: counter indications for the cardiopulmonar stress test or for the use of the device (bone instability, serious contractures, and lower limb vascular disorders), neurological illness (spinal cord injury, multiple schlerosis, and Parkinson’s), lung diseases (COPD), dementia	Lokolift, Hocoma AG + Software LabVIEW (Versión 2009, National Instruments, Austin, TX, USA) (lokomat connected to this software (Hocoma AG, Volketswil, Switzerland))/Visual prescriptive and concurrent (KR) ^a^ Treadmill (h/p/cosmos sports & medical GmbH) Assessment Technology: Ergospirometry (MetaMax 3B, cortex Biophysik GmbH, Leipzig, Germany) Pulsometre (T31, Polar Electro, Kempele, Oulu, Finlandia) + receiver plate (HRMI, Sparkfun, Boulder, CO, USA)	CG (n = 7): RATE + conventional therapy (physiotherapy, speech therapy and conventional therapy) IG (n = 7): idem (except RATE) + FC-RATE Frequency and duration: Sessions of 30´. Three sessions per week for four weeks	Pre-test vs. post-test Significant and effective: None Not significant: Peak Oxygen Uptake (VO_2 PEAK_) absolute (mL Kg/min), VO_2 PEAK_ relative (mL Kg/min), Peak Work Rate (P_PEAK_) (W), Peak Ventilation Rate (VE_PEAK_) (L/min), Peak Respiratory Rate (Rf_PEAK_) (L/min), Peak Heart Rate (HR_PEAK_) (beats/min), Peak Respiratory Exchange Ratio (RER_PEAK_) (VCO_2_/VO_2_)
18. Sungkarat, S.; 2011 [41] To determine whether external feedback to promote symmetrical weight distribution during standing and walking would improve gait performance and balance in people with stroke	N = 35 Sex = 11 female (31.4%); 24 male (68.6%) Age = 53 ± 9.3 years Inclusion criteria: first unilateral stroke with hemiparesis, Orpington Evaluation: 3.2–5.2, gait minimum 10 m with or without help, stable health condition to understand rules and participation Exclusion criteria: comorbidity or complication which impedes gait training, cognitive and/or communicative deterioration, severe leg spasticity, negligence, miss more than 3 sessions	Tecnology I-ShoWS (Insole Shoe Wedge and Sensors) consists of: footswitch for non-paretic foot with acoustic feedback during swing phase Lateral wedge insole of 7° in non-paretic foot to force change of weight in the paretic foot Pressure switch on paretic foot with acoustic feedback about weight bearing during stance fase of this foot (if weight is exceeded) (Pedal actuator/Acoustic descriptive and terminal (KR) (Pressure sensor/Acoustic descriptive and terminal (KR) Assessment Technology: GAITRite Electronic walkway system (CIR systems Inc., Clifton, NJ, USA)	CG (n = 18): programme of conventional retraining IG (n = 17): readaptation of gait using a wedge as an insole and set-up sensors (I-ShoWS). Frequency and duration: 15 sessions of 60 min for five days a week. Each session divided into 30 min gait retraining and the other 30 min for other conventional rehabilitation treatments	Pre-test vs. post-test Significant and effective: Gait Speed (cm/s), Step Length Asymmetry Ratio (m), Single Support Time Asymmetry Ratio (sec), Berg Balance Scale (points), Timed Up and Go (sec), Loading on Paretic Leg during Stance (%body weight) Not significant: None
19. Won et al., 2015 [42] Effects of a novel walking training program with postural correction and visual feedback on walking function in patients with post-stroke hemiparesis	N = 16 Sex = 8 female (50%); 8 male (50%) Age = 60.35 ± 15.35 years Inclusion criteria: Stroke more than 6 months ago, Mini-mental test > 25, without orthopedic or cardiopulmonary problems, and with no psychological or emotional disorders Exclusion criteria: Not specified	Rear camera presenting body alignment in the coronal plane and load cells incorporated in a base plate under the treadmill (FTS)^®^/Visual prescriptive and concurrent (KP) Assessment Technology: Functional Training System, Marpe Co., Ltd., Jeonju, Korea	CG (n = 8): functional recovery of gait IG (n = 8): idem + postural correction using elastic bands + visual feedback during gait Frequency and duration: 30 min walking, twice a day for two weeks (speed adjusted to 2–4 m/s)	Pre-test vs. post-test Significant and effective: Step Length Ratio, Step Time Ratio, Stride Length (cm), Stance Phase Ratio, Swing Phase Ratio, 10-m Walk Test (10MWT) (sec) Not significant: None
20. Zanotto, D. et al., 2013 [10] To investigate whether the most commonly used combination of feedback (i.e., haptic and visual) could be either enhanced by adding acoustic feedback or successfully substituted with a combination of kinetic guidance and acoustic feedback	N = 32 Sex = 12 female (37.5%); 20 male (62.5%) Age = 24.7 ± 3.8 years Inclusion criteria: right handed, without musculoskeletal or neurological problems Exclusion criteria: Not specified	ALEX II^®^: Exoesqueleto + Software + pressure sensor (interlink electronic FSR 4065) in the shoe + Speakers + Real time controller (PPC DS1103 controller Board2, dSPACE GmbH, Paderborn, Germany): Acoustic prescriptive and terminal (KR) Acoustic descriptive and concurrent (KP) Visual prescriptive and concurrent (KP) Assessment Technology: Load cells built into a baseplate under the walking belt of the treadmill	Kinetic guidance (robot) CG (n = 8): visual feedback (board) that shows a way next to the ankles IG I (n = 8): complex and continuous acoustic feedback (information of gait performance) IG II (n = 8): simple acoustic feedback by pressure sensor that produces a “beep” to mark the step. IG III (n = 8): visual feedback (CG) in combination with simple acoustic feedback (IG II) Frequency and duration: not specified	Pre-test vs. Post-test Normalized Error Area (NEA): Significant and effective: IG II and IG III Not significant: IG I NEA stance: Significant and effective: IG I Not significant: IG II and IG III NEA early swing: Significant and effective: IG I, IG II and IG II Not significant: None NEA late swing: Significant and effective: IG I, IG II and IG III Not significant: None ROM x: Significant and effective: IG II and IG III Not significant: IG I ROM y: Significant and effective: IG II and IG III Not significant: IG I Normalized Error in Stride Period (Terr): Significant and effective: IG I, IG II, IG III Not significant: None Stance Time Period (STP) ratio: Significant and effective: None Not significant: IG I, IG II and IG III

^1^ N = Total Sample; ^2^ KP = Knowledge of Performance; ^3^ KR = Knowledge of Result; ^4^ CG = Control Group; ^5^ IG = Intervention Group; ^6^ CI = Confidence Interval; ^7^ ES = Effect Size; ^a^ Additional Technology. (*) The word “significant” means statistically significant. Therefore, “not significant” means that the outcomes of the study were not statistically significant. “Significant and effective” means that the outcomes show a significant effect of the technology-based feedback in improving the parameters indicated. “Significant and not effective” means significantly not effective in improving the parameters indicated.

**Table 4:** Completed PEDro quality appraisal.

Study	Criteria											Total Score
	1	2	3	4	5	6	7	8	9	10	11	
1. Baram, Y. et al., 2012 [17]	X	✓	X	✓	X	X	X	✓	✓	X	X	4
2. Brasileiro, A. et al., 2015 [18]	X	✓	X	✓	X	X	X	✓	✓	✓	✓	6
3. Byl, N. et al., 2015 [19]	✓	✓	X	X	X	X	X	✓	X	✓	✓	4
4. Drużbicki, M. et al., 2015 [5]	✓	✓	✓	✓	X	X	✓	✓	✓	✓	✓	8
5. El-Tamawy, M. et al., 2012 [23]	✓	✓	X	✓	X	X	✓	✓	X	✓	✓	6
6. Fu, M.C. et al., 2014 [26]	✓	✓	X	X	X	X	X	✓	✓	✓	X	4
7. Ginis, P. et al., 2016 [38]	✓	✓	X	✓	X	X	X	✓	✓	✓	✓	6
8. Hunt, M.A. et al., 2014 [20]	✓	✓	X	✓	X	X	X	✓	✓	✓	✓	6
9. Jung, K. et al., 2015 [7]	✓	✓	✓	✓	X	X	✓	✓	X	✓	✓	7
10. Khallaf, M.E. et al., 2014 [39]	✓	✓	✓	✓	X	X	X	X	X	✓	✓	5
11. Ki, K. et al., 2015 [21]	✓	✓	X	✓	X	X	X	✓	X	✓	✓	5
12. Lipsitz, L.A. et al., 2015 [22]	✓	✓	✓	X	X	X	✓	X	X	✓	X	4
13. Ochi, M. et al., 2015 [14]	✓	✓	✓	✓	X	X	✓	X	X	✓	✓	6
14. Quinzaños Fresnedo, J. et al., 2015 [15]	✓	✓	X	✓	X	X	X	✓	✓	✓	✓	6
15. Segal, N.A. et al., 2015 [11]	✓	✓	✓	✓	X	X	X	X	X	✓	✓	5
16. Shen, X. et al., 2014 [40]	✓	✓	X	✓	X	X	✓	✓	✓	✓	✓	7
17. Stoller, O. et al., 2015 [16]	✓	✓	✓	✓	X	X	✓	X	✓	✓	✓	7
18. Sungkarat, S. et al., 2011 [41]	✓	✓	✓	✓	X	X	✓	✓	X	✓	✓	7
19. Won, S.H. et al., 2015 [42]	✓	✓	✓	✓	X	X	X	✓	✓	✓	✓	7
20. Zanotto, D. et al., 2013 [10]	✓	✓	X	✓	X	X	X	✓	✓	✓	✓	6

Criteria: ^1^ Eligibility criteria were specified (not used for score); ^2^ Subjects were randomly allocated to groups; ^3^ Allocation was concealed; ^4^ Groups were similar at baseline regarding the most important prognostic indicators; ^5^ There was blinding of all subjects; ^6^ There was blinding of all therapists who administered the therapy; ^7^ There was blinding of all assessors who measured at least one key outcome; ^8^ Measures of at least one key outcome were obtained from more than 85% of the subjects initially allocated to groups; ^9^ All subjects for whom outcome measures were available received the treatment or control condition as allocated or, where this was not the case, data for at least one key outcome was analyzed by ‘intention-to-treat’; ^10^ The results of between-group statistical comparisons are reported for at least one key outcome; ^11^ The study provides both point measures and measures of variability for at least one key outcome). ✓ = criteria met; X = criteria not met.

**Table 5:** Outline of the types of feedback used in each study.

	Feedback	Knowledge Performance	Knowledge Result	Concurrent/Immediate	Terminal/Retarded	Descriptive	Prescriptive
1. Baram, Y.; 2012 [17]	Visual	X		X			X
	Acoustic		X		X	X	
2. Brasileiro, A. et al., 2015 [18]	Visual	X		X			X
3. Byl et al., 2015 [19]	Visual	X		X			X
4. Druzbicki, M. et al., 2015 [5]	Visual	X		X			X
	Acoustic		X		X	X	
5. El-Tamawy et al., 2012 [23]	Haptic		X	X			X
6. Fu, M.C. et al., 2014 [26]	Haptic		X		X	X	
7. Ginis, P. et al., 2016 [38]	Acoustic	X		X		X	
8. Hunt et al., 2014 [20]	Visual	X		X			X
9. Jung et al., 2015 [7]	Acoustic		X		X	X	
10. Khallaf et al., 2014 [39]	Visual		X		X	X	
11. Ki et al., 2015 [21]	Acoustic		X		X	X	
12. Lipsitz, L.A. et al., 2015 [22]	Haptic		X		X	X	
13. Ochi et al., 2015 [14]	Visual		X	X			X
14. Quinzaños Fresnedo, J.; 2015 [15]	Acoustic		X		X	X	
15. Segal, N.; 2015 [11]	Visual		X	X			X
16. Shen, X.; 2014 [40]	Visual	X			X	X	
	Visual	X		X			X
17. Stoller, O. et al., 2015 [16]	Visual		X	X			X
18. Sungkarat, S.; 2011 [41]	Acoustic		X		X	X	
	Acoustic		X		X	X	
19. Won et al., 2015 [42]	Visual	X		X			X
20. Zanotto, D. et al., 2013 [10]	Acoustic		X		X		X
	Acoustic	X		X		X	
	Visual	X		X			X

**Table 6:** Interventions with technology-based feedback and their effectiveness in improving gait parameters.

	Feedback	Walking Speed (m/s)	Stride Length (m)	Cadence (steps/min)	TUG (s)	Berg Balance	10MWT (m/s)	2MWT (m)	ROM Hip (°)	ROM Knee (°)	ROM Ankle (°)
1. Baram, Y.; 2012 [17]	Visual	X	X								
	Acoustic	X	X								
2. Brasileiro, A. et al., 2015 [18]	Visual	X	X	X					X	X	X
3. Byl et al., 2015 [19]	Visual	X	X		X	X					
4. Druzbicki, M. et al., 2015 [5]	Visual	X	X	X	X						
	Acoustic	X	X	X	X						
5. El-Tamawy et al., 2012 [23]	Haptic	X	X	X					X	X	X
6. Fu, M.C. et al., 2014 [26]	Haptic										
7. Ginis, P. et al., 2016 [38]	Acoustic	X	X					X			
8. Hunt et al., 2014 [20]	Visual										
9. Jung et al., 2015 [7]	Acoustic	X									
10. Khallaf et al., 2014 [39]	Visual										
11. Ki et al., 2015 [21]	Acoustic				X						
12. Lipsitz, L.A. et al., 2015 [22]	Haptic	X			X						
13. Ochi et al., 2015 [14]	Visual	X									
14. Quinzaños-Fresnedo, J.; 2015 [15]	Acoustic	X		X							
15. Segal, N.; 2015 [11]	Visual										
16. Shen, X.; 2014 [40]	Visual (AT *)	X	X								
	Visual (3m *^1^)	X	X								
	Visual (6m)	X	X								
17. Stoller, O. et al., 2015 [16]	Visual										
18. Sungkarat, S.; 2011 [41]	Acoustic	X			X	X					
19. Won et al., 2015 [42]	Visual		X				X				
20. Zanotto, D. et al., 2013 [10]	Acoustic										
	Visual										

X = Parameter measured; Significant and effective = 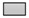; Significant and not effective = 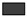; Not significant = 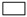; * After Treatment; * ^1^ months; TUG = Test Up and Go; 10MWT = 10 meters Walk Time; 2MWT = 2-min test; ROM = Range Of Motion.

## Abbreviations

10MWT	10-m Walk Test
ABC	Activities-Specific Balance Confidence
ADL	Activities of Daily Living
AFO	Ankle Foot Orthosis
CG	Control Group
CI	Confidence Interval
COPD	Chronic Obstructive Pulmonary Disease
CP	Cerebral Palsy
CT	Computed Tomography
ES	Effect Size
FAC	Functional Ambulation Classification
FC-RATE	Feedback Controlled Robotics Assisted Treadmill Exercise
FIM™	Functional Independence Measure
FTS^®^	Functional Training System
FTSTS test	Five Times Sit To Stand
GAGT	GAR-Assisted Gait Training Group
GAR	Gait-Assistance Robot
GCB	“Bathroom Scale” Training Group
GCV	“Verbal Instruction” Training Group
GFB	“Haptic Biofeedback” Training Group
HRpeak	Peak Heart Rate
IG	Intervention Group
IQR	Barthel Index
IT	Information Technology
KOOS	Knee Injury and Osteoarthritis Outcome Score
KP	Knowledge of Performance
KR	Knowledge of Result
LCnp	Length of the Cycle of Non-Paretic Limb
LCp	Length of the Cycle of Paretic Limb
LDCW	Long Distance Corridor Walk
LLFDI	Late Life Function and Disability Index
LOS	Limit Of Stability
MAS	Modified Ashworth Scale
MRI	Magnetic Resonance Imaging
N	Total Sample
NEA	Normalized Error Area
OA	Osteoarthritis
OCGT	Overground Conventional Gait Training Group
Ppeak	Peak Work Rate
PBWS	Partial Body Weight Supported
PD	Parkinson´s Disease
RATE	Robotics Assisted Treadmill Exercise
RCTs	Randomised Controlled Trials
RERpeak	Peak Respiratory Exchange Ratio
Rfpeak	Peak Respiratory Rate
ROM	Range of Movement
SD	Standar Deviation
STFnp	Stance Phase of the Non-Paretic Limb
STFp	Stance Phase of the Paretic Limb
STP	Stance Time Period
SWFnp	Swing Phase of the Non-Paretic Limb
SWFp	Swing Phase of the Paretic Limb
Terr	Normalized Error in the Stride Period
TUG test	Timed Up and Go
UPDRS	United Parkinson´s Disease Rating Scale
VEpeak	Peak Ventilation Rate

## References

[1] Chamorro-Moriana G., Ridao-Fernández C., Ojeda J., Benítez-Lugo M., Sevillano J.L. (2016). Reliability and validity study of the Chamorro Assisted Gait Scale for people with sprained ankles, walking with forearm crutches. PLoS ONE.

[2] Chamorro-Moriana G., Rebollo-Roldán J., Jiménez-Rejano J.J., Chillón-Martínez R., Suárez-Serrano C. (2013). Design and validation of GCH System 1.0 which measures the weight-bearing exerted on forearm crutches during aided gait. Gait Posture.

[3] Whittle M.W. (2003). Gait Analysis: An Introduction.

[4] Van Den Noort J.C., Steenbrink F. (2015). Real time visual feedback for gait retraining: Toward application in knee osteoarthritis. Med. Biol. Eng. Comput..

[5] Druzbicki M., Guzik A., Przysada G., Kwolek A., Brzozowska-Magoń A. (2015). Efficacy of gait training using a treadmill with and without visual biofeedback in patients after stroke: A randomized study. J. Rehabil. Med..

[6] Agresta C., Hall J. (2015). Gait Retraining for Injured and Healthy Runners using Augmented Feedback: A Systematic Literature Review. J. Orthop. Sports Phys. Ther..

[7] Jung K., Kim Y., Cha Y., In T., Hur Y., Chung Y. (2015). Effects of gait training with a cane and an augmented pressure sensor for enhancement of weight bearing over the affected lower limb in patients with stroke : A randomized controlled pilot study. Clin. Rehabil..

[8] Isakov E. (2007). Gait rehabilitation: A new biofeedback device for monitoring and enhancing weight-bearing over the affected lower limb. Eura Medic..

[9] Basta D., Rossi-Izquierdo M., Soto-Varela A., Greters M.E., Bittar R.S., Steinhagen-Thiessen E., Eckardt R., Harada T., Goto F., Ogawa K. (2011). Efficacy of a vibrotactile neurofeedback training in stance and gait conditions for the treatment of balance deficits: A double-blind, placebo-controlled multicenter study. Otol. Neurotol..

[10] Zanotto D., Rosati G., Spagnol S., Stegall P., Agrawal S.K. (2013). Effects of Complementary Auditory Feedback in Robot-Assisted Lower Extremity Motor Adaptation. IEEE Trans. Neural Syst. Rehabil. Eng..

[11] Segal N.A., Glass N.A., Teran-Yengle P., Singh B., Wallace R.B., Yack H.J. (2015). Intensive Gait Training for Older Adults with Symptomatic Knee Osteoarthritis. Am. J. Phys. Med. Rehabil..

[12] Nanhoe-Mahabier W., Allum J.H., Pasman E.P., Overeem S., Bloem B.R. (2012). The effects of vibrotactile biofeedback training on trunk sway in Parkinson’s disease patients. Parkinsonism Relat. Disord..

[13] Fernández R., Rodríguez B., Barcia B., Souto S., Chouza M., Martínez S. (1998). Generalidades sobre Feedback (o retroalimentación). Fisioterapia.

[14] Ochi M., Wada F., Saeki S., Hachisuka K. (2015). Gait training in subacute non-ambulatory stroke patients using a full weight-bearing gait-assistance robot: A prospective, randomized, open, blinded-endpoint trial. J. Neurol. Sci..

[15] Quinzaños Fresnedo J., Sahagún Olmos R.C., León Hernández S.R., Pérez Zavala R., Quiñones Uriostegui I., Solano Salazar C.J., Cruz Lira R.T., Tinajero Santana M.C. (2015). Efectos a corto plazo del entrenamiento de la marcha en una órtesis robótica (Lokomat^®^) con retroalimentación auditiva en pacientes con lesión medular incompleta crónica. Rehabilitacion.

[16] Stoller O., de Bruin E.D., Schindelholz M., Schuster-Amft C., de Bie R.A., Hunt K.J. (2015). Efficacy of Feedback-Controlled Robotics-Assisted Treadmill Exercise to Improve Cardiovascular Fitness Early After Stroke. J. Neurol. Phys. Ther..

[17] Baram Y., Lenger R. (2012). Gait Improvement in Patients with Cerebral Palsy by Visual and Auditory Feedback. Neuromodulation: Technol. Neural Interface.

[18] Brasileiro A., Gama G., Trigueiro L., Ribeiro T., Silva E., Galvão É., Lindquist A. (2015). Influence of visual and auditory biofeedback on partial body weight support treadmill training of individuals with chronic hemiparesis: A randomized controlled clinical trial. Eur. J. Phys. Rehabil. Med..

[19] Byl N., Zhang W., Coo S., Tomizuka M. (2015). Clinical impact of gait training enhanced with visual kinematic biofeedback: Patients with Parkinson’s disease and patients stable post stroke. Neuropsychologia.

[20] Hunt M.A., Takacs J., Hart K., Massong E., Fechko K., Biegler J. (2014). Comparison of mirror, raw video, and real-time visual biofeedback for training toe-out gait in individuals with knee osteoarthritis. Arch. Phys. Med. Rehabil..

[21] Ki K.I., Kim M.S., Moon Y., Choi J.D. (2015). Effects of auditory feedback during gait training on hemiplegic patients’ weight bearing and dynamic balance ability. J. Phys. Ther. Sci..

[22] Lipsitz L.A., Lough M., Niemi J., Travison T., Howlett H., Manor B. (2015). A shoe insole delivering subsensory vibratory noise improves balance and gait in healthy elderly people. Arch. Phys. Med. Rehabil..

[23] El-Tamawy M., Darwish M., Khallaf M. (2012). Effects of augmented proprioceptive cues on the parameters of gait of individuals with Parkinson′s disease. Ann. Indian Acad. Neurol..

[24] Parker J., Mountain G., Hammerton J. (2011). A review of the evidence underpinning the use of visual and auditory feedback for computer technology in post-stroke upper-limb rehabilitation. Disabil. Rehabil. Assist. Technol..

[25] Thikey H., Grealy M., van Wijck F., Barber M., Rowe P. (2012). Augmented visual feedback of movement performance to enhance walking recovery after stroke: Study protocol for a pilot randomised controlled trial. Trials.

[26] Fu M.C., DeLuke L., Buerba R., Fan R.E., Zheng Y.J., Leslie M.P., Baumgaertner M.R., Grauer J.N. (2014). Haptic biofeedback for improving compliance with lower-extremity partial weight bearing. Orthopedics.

[27] Chamorro-Moriana G., Sevillano J.L., Ridao-Fernández C. (2016). A compact forearm crutch based on force sensors for aided gait: Reliability and validity. Sensors.

[28] Mortensen D.H., Bech S., Begault D.R., Adelstein B.D. (2009). The relative importance of visual, auditory, and haptic information for the user’s experience of mechanical switches. Perception.

[29] Lefmann S., Russo R., Hillier S. (2017). The effectiveness of robotic-assisted gait training for paediatric gait disorders: Systematic review. J. Neuroeng. Rehabil..

[30] Sharma D.A., Chevidikunnan M.F., Khan F.R., Gaowgzeh R.A. (2016). Effectiveness of knowledge of result and knowledge of performance in the learning of a skilled motor activity by healthy young adults. J. Phys. Ther. Sci..

[31] Liberati A., Altman D.G., Tetzlaff J., Mulrow C., Gøtzsche P.C., Ioannidis J.P.A. (2009). The PRISMA statement for reporting systematic reviews and meta-analyses of studies that evaluate health care interventions: Explanation and elaboration. J. Clin. Epidemiol..

[32] Moseley A.M., Herbert R.D., Sherrington C., Maher C.G. (2002). Evidence for physiotherapy practice: A survey of the Physiotherapy Evidence Database (PEDro). Aust. J. Physiother..

[33] Yamato T.P., Maher C., Koes B., Moseley A. (2017). The PEDro scale had acceptably high convergent validity, construct validity, and interrater reliability in evaluating methodological quality of pharmaceutical trials. J. Clin. Epidemiol..

[34] Sherrington C., Herbert R., Maher C., Moseley A. (2000). PEDro. A database of randomized trials and systematic reviews in physiotherapy. Man Ther..

[35] Maher C.G., Sherrington C., Herbert R.D., Moseley A.M. (2003). Reliability of the PEDro scale for rating quality of randomized controlled trials. Phys. Ther..

[36] De Morton N.A. (2009). The PEDro scale is a valid measure of the methodological quality of clinical trials: A demographic study. Aust. J. Physiother..

[37] Moher D., Liberati A., Tetzlaff J., Altman D.G. (2009). Academia and Clinic Annals of Internal Medicine Preferred Reporting Items for Systematic Reviews and Meta-Analyses: The PRISMA Statement. Annu. Intern. Med..

[38] Ginis P., Nieuwboer A., Dorfman M., Ferrari A., Gazit E., Canning C.G., Rocchi L., Chiari L., Hausdorff J.M., Mirelman A. (2016). Feasibility and effects of home-based smartphone-delivered automated feedback training for gait in people with Parkinson’s disease: A pilot randomized controlled trial. Parkinsonism Relat. Disord..

[39] Khallaf M.E., Gabr A.M., Fayed E.E. (2014). Effect of Task Specific Exercises, Gait Training, and Visual Biofeedback on Equinovarus Gait among Individuals with Stroke: Randomized Controlled Study. Neurol. Res. Int..

[40] Shen X., Mak M.K.Y. (2014). Balance and Gait Training with Augmented Feedback Improves Balance Confidence in People with Parkinson’s Disease. Neurorehabil. Neural Repair.

[41] Sungkarat S., Fisher B.E., Kovindha A. (2011). Efficacy of an insole shoe wedge and augmented pressure sensor for gait training in individuals with stroke: A randomized controlled trial. Clin. Rehabil..

[42] Won S.H., Kim J.C., Oh D.W. (2015). Effects of a novel walking training program with postural correction and visual feedback on walking function in patients with post-stroke hemiparesis. J. Phys. Ther. Sci..

[43] Tzetzis G., Votsis E., Kourtessis T. (2008). The effect of different corrective feedback methods on the outcome and self confidence of young athletes. J. Sports Sci. Med..

[44] Sardini E., Serpelloni M., Lancini M. (2015). Wireless Instrumented Crutches for Force and Movement Measurements for Gait Monitoring. IEEE Trans. Instrum. Meas..

[45] Tuttle N., Jacuinde G. (2011). Design and Construction of a Novel Low-Cost Device to Provide Feedback on Manually Applied Forces. J. Orthop. Sport Phys. Ther..

[46] Winstein C.J., Pohl P.S., Cardinale C., Green A., Scholtz L., Waters C. (1996). Learning a partial-weight-bearing skill: Effectiveness of two forms of feedback. Phys. Ther..

[47] Warren C.G., Lehmann J. (1975). Training procedures and biofeedback methods to achieve controled partial weight bearing: An assessment. Arch. Phys. Med. Rehabil..

[48] Salmoni A.W., Schmidt R.A. (1984). Knowledge of results and motor learning: A review and critical reappraisal. Psychol. Bull..

[49] Sigrist R., Rauter G., Riener R., Wolf P. (2013). Augmented visual, auditory, haptic, and multimodal feedback in motor learning: A review. Psychon. Bull. Rev..

[50] Sülzenbrück S., Heuer H. (2011). Type of visual feedback during practice influences the precision of the acquired internal model of a complex visuo-motor transformation. Ergonomics.

[51] Li J., Wu T., Xu Z., Gu X. (2014). A pilot study of post-total knee replacement gait rehabilitation using lower limbs robot-assisted training system. Eur. J. Orthop. Surg. Traumatol..

[52] Kloos A.D., Kegelmeyer D.A., White S. (2012). The impact of different types of assistive devices on gait measures and safety in Huntington’s disease. PLoS ONE.

[53] Thomas K.S., Russell D.M., Van Lunen B.L., Colberg S.R., Morrison S. (2017). The impact of speed and time on gait dynamics. Hum. Mov. Sci..

[54] Figueiredo P.R.P., Silva P.L.P., Avelar B.S., Chagas P.S.C., Oliveira L.C.P., Mancini M.C. (2013). Assessment of gait in toddlers with normal motor development and in hemiplegic children with mild motor impairment: A validity study. Brazilian J. Phys. Ther..

[55] Yang C.C., Hsu Y.L., Shih K.S., Lu J.M. (2011). Real-time gait cycle parameter recognition using a wearable accelerometry system. Sensors.

